# Veterinary-Prescribed Physical Activity: Feasibility and Acceptability among Veterinary Staff and Dog Owners

**DOI:** 10.3390/ijerph18052339

**Published:** 2021-02-27

**Authors:** Katrina Oselinsky, Colleen G. Duncan, Heather E. Martinez, Dan J. Graham

**Affiliations:** 1 Department of Psychology, Colorado State University, 1876 Campus Delivery, Fort Collins, CO 80523, USA; dan.graham@colostate.edu; 2 Department of Microbiology, Immunology and Pathology, Colorado State University, 1619 Campus Delivery, Fort Collins, CO 80523, USA; colleen.duncan@colostate.edu; 3 Council of State and Territory Epidemiologists, Colorado Department of Public Health and Environment, 4300 Cherry Creek S Dr, Denver, CO 80246, USA; heather.h.martinez@state.co.us

**Keywords:** exercise prescription, physical activity, dog-walking, veterinary, One Health

## Abstract

Physical inactivity remains a global epidemic leading to an estimated 5 million preventable deaths per year. Although there exist numerous public-health campaigns aimed at increasing physical activity (PA), a potentially fruitful but underexplored avenue to promote both human and animal health is veterinary-prescribed PA programs. The aim of this study was to determine the feasibility and acceptability of incorporating veterinary-prescribed PA programming into a diverse array of clinic settings. Participants (n = 722 veterinary-clinic staff (VS); n = 1028 dog owners (DOs)) completed an online survey assessing: (a) the perceived importance of PA for promoting health and preventing disease, (b) willingness to participate in a veterinary-prescribed PA program, and (c) potential benefits and barriers of such a program. Both groups of participants indicated that PA is important for both human and animal health (97% and 98% of VS and 92% and 93% of DOs said PA is very or extremely important for animal and human health, respectively). Additionally, most participants in both groups expressed an interest in participating in a veterinary-prescribed PA program in the future, with only 11% of DOs and 10% of VS saying they were not interested. Benefits and barriers of this type of intervention for both practitioners and patients were also identified. Incorporating veterinary-prescribed PA programming into veterinary clinics seems to be acceptable to both DOs and VS. Additionally, many VS believe such programming would be feasible at their clinics; thus, pursuing such programs appears to be a promising avenue for promoting human and animal health.

## 1. Introduction

Physical inactivity has become a worldwide epidemic. Globally, 1 in 4 adults do not meet the World Health Organization’s recommended levels of physical activity (PA), resulting in 5 million preventable deaths per year [1]. Despite the best efforts of numerous public-health campaigns aimed at increasing PA [2,3,4], physical inactivity remains one of the most pervasive modifiable risk factors for developing chronic disease [5,6]. Although the evidence is mixed, PA prescriptions have shown promise in increasing activity [5,7,8]. Due to the ability of PA prescriptions to alter patient behavior [5,9,10], it has been recommended that PA prescriptions become a standard of clinical care [5,9]. Although prescribing PA offers great potential, many physicians and general practitioners are hesitant to discuss PA with their patients due to a lack of knowledge, training, and time [11,12]. Additionally, weight-related discussions between healthcare providers and patients vary based on both patient and provider characteristics, including weight status [13]. Many practitioners (e.g., those who are insufficiently active and/or overweight themselves) are reluctant to discuss PA with patients, particularly given stigma associated with overweight/obesity [12,13,14,15]. In addition, some communities do not have convenient or regular access to physicians, limiting the scalability of physician-prescribed PA interventions. It is therefore essential to identify other entities within the community who have the ability to deliver PA prescriptions.

Dog-walking interventions, and more specifically, veterinarian-prescribed PA programs, have been successful at increasing companion-animal PA [16,17]. A common result of increased companion-animal PA is increased human PA. Research consistently demonstrates that dog owners (DOs) engage in more leisure-time walking and are more likely than non-DOs to meet or exceed PA recommendations [17,18,19,20]. Additionally, DOs are less likely to allow common barriers to PA (e.g., lack of time, motivation, or poor weather) to reduce their engagement [20]. A recent interview study examining reasons why DOs have higher PA adherence than do non-DOs reported that a perceived obligation to a pet was one of the most common reasons people chose to engage in consistent PA [20]. Results suggest that this sense of obligation to a pet is powerful enough to circumvent commonly reported barriers such as bad weather or lack of time. Additionally, the researchers found that DOs were willing to put the PA and health needs of their pet before their own [20]. This meant that DOs were willing to sacrifice their time and their own activity preferences to prioritize those of their pet. Dogs also provide consistent social support, an essential element of adoption and maintenance of PA [21,22]. Finally, dog-walking has the potential to advance a One Health agenda. One Health is the idea that human, animal, and environmental health are interrelated [23]. Since health is determined by many factors, researchers must work together to craft transdisciplinary solutions to our most pressing health problems. Dog-walking may be one such solution to problems related to not only the health of the humans and dogs who are walking together, but also the health of the natural environment. Research has shown that people who spend time in nature (in this case, through increased dog-walking) are more inclined to protect it, suggesting that environmental benefits, like human and animal health benefits, can be realized through dog-walking interventions [23]. Despite these forces of obligation and support, many dog owners still do not walk their dogs regularly.

Although dog-walking leads to positive health benefits for both humans and animals, there is a large discrepancy between the number of people who own dogs and the number who regularly walk their dogs. For example, 63.4 million Americans own dogs; however, only 60% routinely walk their dogs [16,17]. Research by Baumann et al. [24,25] suggests that in Australia, PA from dog-walking alone could reduce the incidence of human chronic disease by 5–9% should all DOs consistently walk their pets [20]. This commitment to regular dog walking would result in estimated healthcare savings of USD 175 million [20]. This suggests that there are substantial health and financial benefits that can be realized through increased dog walking. Although DOs have been shown to follow veterinary advice [16,20], few studies have examined the efficacy of veterinary-prescribed PA interventions for improving both human and animal health. Research by Duncan et al. [16], Kushner et al. [21], and Byers et al. [26] evaluated the impact of veterinary-prescribed PA programs on both human and animal health. Although all three studies concluded that veterinary-prescribed PA interventions can benefit both human and companion animal health, they were not without their limitations.

Research by Duncan et al. [16] was conducted in an academic, veterinary teaching hospital that has a variety of differences from many private veterinary practices. First, there was no fee associated with participation in this study, thus limiting the researchers’ ability to discern if DOs would be willing to pay for this type of service. Additionally, those administering the PA prescriptions were part of the study team and were therefore motivated to provide this service to their clients. It is possible that private-practice clinic staff who are not researchers and who did not design the program may be less enthusiastic about delivering a similar intervention. Additionally, the work of Kushner et al. [21] and Byers et al. [26] restricted their samples to only overweight dogs, owners, or dog–owner pairs. In order to determine the feasibility and acceptability of veterinary-prescribed PA interventions for public-health promotion among private-practice veterinary staff and their clients, more work must be conducted to determine: a) the willingness of veterinary staff to offer this service to their clients, and b) the interest of DOs in paying for/utilizing this service.

To address these gaps in the research literature, this study examines the feasibility and acceptability of implementing a veterinary-prescribed PA program to enhance both human and animal health. This exploratory study sought to determine the level of interest and perceived benefits and barriers of implementing a novel PA program for both dogs and humans into private-practice veterinary clinics from both DOs and clinic staff.

## 2. Materials and Methods

### 2.1. Study Design

This study was comprised of an online survey administered to both veterinary staff (i.e., veterinarians, veterinary technicians, veterinary assistants, and individuals working in client services at veterinary clinics) and DOs at least 18 years of age. The research team obtained approval from its institutional review board prior to data collection, and all participants were required to provide informed consent before engaging in survey activities.

### 2.2. Participants

Data were collected in the fall of 2019. A convenience sample of 766 veterinary staff (VS) and 1067 DOs completed online surveys (44 veterinary participants and 39 dog owners were removed due to incomplete data or for failing to meet the inclusion criteria of either owning a dog (for DOs) or working in veterinary medicine (for VS)), resulting in samples of 722 VS and 1028 DOs. Veterinary personnel were recruited through a paid marketing email and a Facebook post associated with a veterinary magazine. DOs who were 18 years of age or older were recruited through Amazon’s Mechanical Turk (MTurk) and were compensated USD 2 for completing the 26-item (approximately 5-minute) survey. Veterinary personnel who completed the survey were entered into a raffle to win 1 of 10 USD 50 Amazon gift cards.

### 2.3. Procedures

After reading an overview of the study purpose, participants provided informed consent before continuing to the survey questions. After completing the survey questions, DOs received a compensation code in MTurk to receive their USD 2 payment, and VS entered a separate portal to provide contact information to be entered into the random drawing to win 1 of the 10 USD 50 gift cards.

### 2.4. Measures

#### 2.4.1. Variables Assessing Feasibility and Acceptability of Veterinary-Prescribed Exercise

The full text of survey items along with response options are presented in the Supplementary Materials Appendix A (DO survey) and Appendix B (VS survey). The surveys used in this research were created for the present study and were not assessed for reliability and validity.

Questions for Dog Owners Only: DOs were asked questions to gauge their beliefs and attitudes regarding health and its relationship to PA in both dogs and people. They were also asked to self-report their own PA and the percent of their reported daily PA spent with their dog(s). DOs were also asked questions about their current experiences with their veterinarian regarding PA and whether they believed that increasing PA for their dog(s) would also increase their own PA.

The pilot dog-walking program conducted at Colorado State University’s Veterinary Teaching Hospital was described briefly (Supplementary Appendix A), then DOs were asked whether they would be willing to participate in health-screening activities at their own veterinary clinic like those included in the CSU study. Follow-up items asked DOs about their comfort in receiving specific potential offerings that a veterinary clinic could provide for humans (e.g., height, weight, blood pressure). DOs were also asked which general health-promotion topics that apply to both humans and animals they would be interested in learning more about at a veterinary clinic. Finally, DOs were asked to identify reasons why they might participate in a veterinary-prescribed exercise program and also to identify reasons why they might not, reporting barriers in place that would prevent their involvement.

Questions for Veterinary Staff Only: VS were asked several questions about their job title, responsibilities, and activities, including questions specific to PA-related recommendations and discussions with client owners. Veterinary personnel were asked to identify barriers that reduce their own likelihood of discussing PA during appointments. Like DOs, VS also answered questions to assess their beliefs and attitudes about health and its relationship to PA in both dogs and people.

As in the DO survey, the pilot program conducted at CSU’s Veterinary Teaching Hospital also was described briefly to VS participants (Supplementary Appendix B). Following this description, VS were asked questions about which resources might help them promote healthy behaviors for clients and their pets, which topics would be appropriate to discuss with clients, and what benefits and barriers they perceived related to including basic human-health-promotion services at their clinic. Vet respondents were also asked which groups of clients would be receptive to receiving human-health-promotion information from their veterinarian and which member(s) of the clinical team would be best suited to manage such programming. The final question in the veterinary survey asked whether the respondent or their colleagues would be interested in potentially participating in future initiatives on how to incorporate shared human–animal health initiatives in private practice.

#### 2.4.2. Demographic Data

Both VS and DOs self-reported their age and zip code. DOs also reported their gender and highest level of education completed.

### 2.5. Analyses

Descriptive statistical analyses (means and standard deviations by group) were conducted in 2020 using RStudio Statistical Software version 3.6.1 [21] in order to ascertain the perceived feasibility and acceptability of veterinary-prescribed exercise programs among both those administering such programs (i.e., veterinary personnel) and those receiving the exercise prescriptions (i.e., DOs).

## 3. Results

A total of 1028 DOs and 722 veterinary staff (VS) representing all 50 U.S. states and Washington, DC, completed the respective surveys. The average DO was 35.5 years old (SD = 10.4 years) and the majority were male (60%; 40% female). The median age range of the VS was 30–39 years (min = 20–29; max = 70+). VS came from a variety of clinic settings. The majority (80.3%) worked in small-animal clinical practice, 9.6% worked in mixed-animal clinical practice, 1.7% worked in large-animal clinical practice, 2.6% were from academia, and 5.7% worked in another setting.

### 3.1. Dog Owner (DO) Survey Results

**Figure 1:**
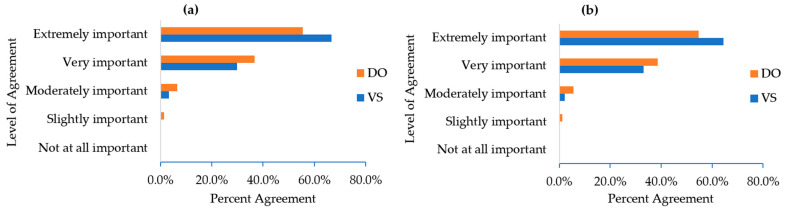
Dog owner (DO; n = 1028) and veterinary staff (VS; n = 722) ratings of the importance of physical activity for (**a**) dog health and (**b**) human health.

Among DOs, 62% reported owning 1 dog, 29% had 2 dogs, and 9% had 3 or more dogs. A high school degree or less had been obtained by 11% of DOs, while 18% reported some college, 12% had an associate’s degree, 47% a bachelor’s degree, and 13% a graduate/professional degree. DO (and VS) beliefs about the importance of regular PA for the health of both dogs and humans are presented in Figure 1. DO (and VS) beliefs about whether PA is useful for preventing and/or treating various health conditions in humans and dogs are presented in Table 1. DO (and VS) beliefs about the percent of people and dogs who would benefit from increasing their PA are presented in Table 2. DO (and VS) reports of the frequency of discussions about the importance of PA for promoting dogs’ health occurring at their veterinary appointments are presented in Table 3.

About a quarter of DOs (24%) reported engaging in 16–30 min of PA per day; the same percentage of DOs (24%) reported 31–45 min/day; a third similarly-sized group (27%) reported 46–60 min/day; 18% reported >60 min/day, and 8% reported ≤15 min/day. Of these daily PA minutes, about a quarter of participants (24%) reported doing 1–20% with their dog; another quarter (25%) completed 21–40% of their daily PA with their dog; a third group of similar size (26%) completed 41–60% of their PA with their dog; 20% reported doing more than 61% of their PA with their dog, and 4% reported doing none of their PA with their dog.

Most DOs (88%) reported that they would make sure their dog was more active on most days or every day if their veterinarian said that increasing the dog’s PA was important for their health; 11% said they would increase their dog’s PA on some days, and 1% of DOs said they would not increase their dog’s PA. Nearly all DOs (93%) indicated that increasing PA for their dog(s) would probably or definitely increase their own PA, while only 2.5% said probably or definitely no; the remaining 4.3% of DOs were unsure how increasing PA for their dog(s) would affect their own PA.

The majority of DOs (73%) said that they would probably or definitely be willing to participate in health-screening activities at their veterinary clinic like those included in the pilot program conducted at CSU, while 17% of DOs were unsure if they would participate in such activities, and 11% said they would probably or definitely not participate. DOs’ comfort with having various biometric markers measured at a veterinary clinic is presented in Table 4. The percent of DOs endorsing reasons why they might and might not participate in joint human–animal activities at private-practice veterinary clinics are reported in Table 5 and Table 6, respectively.

### 3.2. Veterinary Staff (VS) Survey Results

Among the VS respondents, 48% were veterinarians, 36% were veterinary technicians, 7% were veterinary assistants, 4% were involved in patient services, and 5% had a client-service or other role at their veterinary clinic. The largest group of VS (44%) indicated that they were the primary care provider for >75% of their patients; 23% for <25% of their patients, 22% for 51–75% of their patients, and 10% for 26–50% of patients. Responses to the question about the frequency with which VS ask about the patient’s PA when taking a history are presented in Table 3.

Few VS respondents (4%) reported that they recommend additional PA to 0% of their patients; 21% reported recommending additional PA to 1–25% of patients; 33% recommend additional PA to 26–50% of patients; 28% recommend additional PA to 51–75% of patients; 11% recommend additional PA to 76–99% of patients; and 2% of VS reported that they recommend additional PA to 100% of patients.

VS were asked to identify all of the situations in which they recommend increased PA for patients, and 92% reported doing so in cases when the patient is overweight or obese; 81% reported recommending increased PA when the patient displays anxiety/behavior issues; 51% reported doing so when the patient has arthritis; 46% when the patient is healthy; and 36% when the patient has a chronic illness.

About a quarter of VS (26%) indicated that they never highlight the human health benefits of PA within the context of promoting PA for their pets; 38% said that they do so for 1–25% of patients; 18% for 26–50% of patients; 10% for 51–75% of patients; 4% for 76–99% of patients; and 4% of VS noted that they highlight the human health benefits of PA with 100% of their patients.

**Figure 2:**
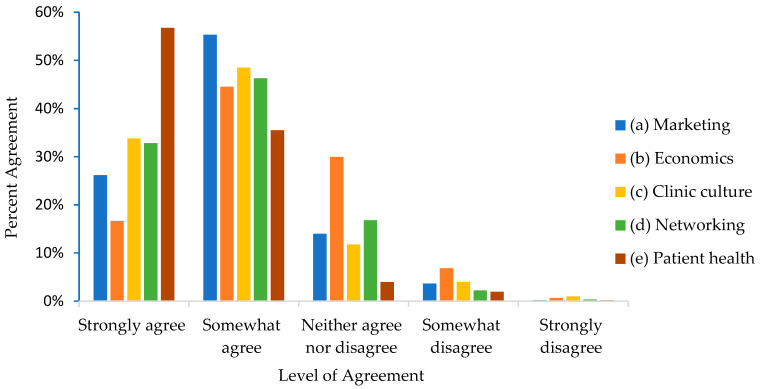
Percent of veterinary staff (n = 631) responding that each of the following would be a beneficial outcome of offering new and unique health programs: (**a**) promotional opportunities; (**b**) increased revenue for their practices; (**c**) would enhance clinic culture; (**d**) would provide networking opportunities; (**e**) could improve the health of clinic patients.

Barriers that VS noted reduce their likelihood of discussing PA during appointments are presented in Table 7. Resources that VS thought might be helpful for promoting health behaviors for clients and their pets are presented in Table 8. The benefits that VS perceived could result from offering human-health-promotion services at their clinic are presented in Figure 2.

VS thought that younger DOs would be the group most receptive to receiving human-health-promotion information from their veterinarian, with 80% of VS saying this client group would be receptive to such information; 56% of VS thought families with children would be receptive; 41% thought senior DOs would be; 38% thought so about DOs with chronic health conditions; 37% about DOs who currently lacked regular access to human health services; while 3% of VS thought that none of their clients would be receptive to this type of information.

Twenty-three percent of VS said that they would be interested in participating in initiatives like those included in the pilot program conducted at CSU; 66% said maybe; and 10% said they would not participate. If such a health-promotion program were to be initiated at their practice, 57% of VS thought that the staff member with the most interest and skills in the area should oversee such initiatives, regardless of their position description; 19% of VS thought that veterinary technicians would be best suited to manage such programming; 10% of VS said veterinarians would be best suited; 8% said the hospital manager; 1% said the clinic owner; 2% said other; and 2% said this was not a topic that should be discussed in a veterinary private-practice setting. VS perceived that lack of time on the part of the clinic staff would be the greatest barrier to implementing such programming, with 79% of VS endorsing this response; 68% also thought a lack of interest among their clients would pose a barrier; 61% thought lack of interest on the part of clinic staff could be a barrier; 39% thought legal concerns could be a barrier; and 38% thought the economic aspects of offering such program could be a barrier.

## 4. Discussion

A large sample of U.S. DOs and veterinary personnel completed online surveys regarding the feasibility and acceptability of veterinary-prescribed PA programs for humans and dogs. Veterinary respondents were representative of the current US veterinary population in terms of age [27]. Respondents to both surveys reported high levels of interest in such programming. There was overwhelming agreement among all those surveyed that PA is “extremely” or “very” important to both dog and human health (DOs = 92% for dogs and 93% for humans; VS = 97% for dogs and 98% for humans). Additionally, respondents to both surveys agreed that the majority of dogs and humans would benefit from increased PA (66% of DOs and 60% of VS indicated that most dogs could benefit from increased PA; 76% of DOs and 78% of VS felt humans would benefit from increased PA). There were also many beneficial outcomes endorsed by VS related to joint human–animal health-promotion programming (Figure 2). Collectively, this work suggests that veterinarians, through PA prescriptions for dog-walking, can have a significant public health impact.

The vast majority of respondents to both surveys showed interest in learning more about human/animal health programming at vet clinics, as only 11% of DOs and 10% of VS indicated they would not be willing to participate in/offer such programs. Additionally, 98% of DOs said they would increase their dog’s PA if instructed to do so by VS. Commensurate with increased canine activity, 93% of DOs said they would increase their own PA if instructed to increase their pet’s PA.

Regarding which types of assessments would be acceptable to DOs, between 70% and 86% of DOs said they would be very or somewhat comfortable with each of the biometric assessments included in the survey (i.e., height, weight, BMI, waist circumference, finger-prick blood test, blood pressure). These results indicate a high degree of DO comfort with having these measurements taken at a veterinary clinic.

Thus, addressing the primary feasibility and acceptability questions these surveys were designed to assess, taken together the present results, indicate that veterinary-prescribed PA programs would be acceptable to many DOs (especially if cost is not prohibitive) as well as to veterinary clinic staff (especially if supportive resources such as program content, implementation, and legal information are provided). The veterinary respondents also indicated that such programs could be feasible for them to conduct, with only a small minority of veterinary respondents indicating that they perceived a lack of interest in such programming among the staff members at their clinic. Encouragingly, most VS believed that any of their clinic’s sufficiently motivated and skilled staff members, regardless of job title, would be suited to delivering such programming.

Beyond feasibility and acceptability, the present results also revealed that PA prescriptions could be indicated for many situations in which PA is not currently a common recommendation provided by VS. Survey responses from VS indicate that they are primarily recommending PA when canine disease is already present. Less than half of VS recommend increased PA to healthy patients, and less than half of clients are getting consistent recommendations to increase PA, despite more than half of dogs in the U.S. being overweight [8]. In addition, with nearly two-thirds of VS never or rarely discussing human health benefits of PA when discussing pet PA, a veterinary-prescribed PA program has the potential to not only improve health outcomes for a significant number of pets, but also to prevent health conditions from arising for pets as well as among owners for whom chronic illness is not yet present.

Results indicated that the belief that PA can help to prevent or treat cancer is much more common among DOs than it is among VS. For example, 15% of DOs reported that PA would help neither people nor dogs with regard to treating or preventing cancer, whereas 32% of VS shared this belief. Conversely a substantially larger proportion of VS respondents (98%) thought that PA would help treat or prevent musculoskeletal disease compared with DOs (82%). PA has been documented to provide preventative and treatment benefits for both of these types of chronic disease [23,24,25]. These results suggest that some additional education for both DOs and VS regarding the benefits of PA could be incorporated into health-promotion programming.

Another related result indicated that DOs and VS may have different perceptions of how frequently PA is discussed in the clinic. Results of this study indicate that VS believe these conversations happen more frequently than do DOs. It should be noted that the samples of DOs and VS who completed these surveys were independent (i.e., these results are not presenting the manner in which two parties to the same conversations recalled or understood these discussions). It is entirely possible that the individuals who responded to our surveys were more invested in PA than the average DOs and VS, and therefore participating DOs and VS may not represent all DOs and VS in the U.S. Nevertheless, it is also possible that VS believe they engage in more discussions regarding PA than they actually do. It is likewise possible that DOs do not attend fully to such discussions every time they occur.

The top three barriers to participating in a veterinary-prescribed PA program reported by DOs were the cost of such programming, the training veterinary staff receive in relation to human health, and the time that receiving such programming could add to veterinary appointments. For VS respondents, the most commonly endorsed barriers were pets having other medical issues, client disability, and lack of client receptivity to PA recommendations. Recommendations to address these barriers are presented below.

Top barriers reported by DOs:
  1. Cost: Although the most commonly cited barrier, it is not clear that the costs of these types of services would be significant (indeed some, such as blood-pressure assessments are currently offered freely at many pharmacies), and costs would likely vary by veterinary clinic. To accurately address potential financial barriers, an economic assessment must be included in future pilot studies.
  2. Training of VS related to human health: To reassure DOs and to assist VS, premade programming materials/resources detailing human-health parameters should be incorporated into any human-health-promotion efforts delivered by VS. VS do not diagnose or make individually targeted recommendations for DOs, which would not change, regardless of adoption and use of this type of programming. Rather, VS would provide general information and encourage DOs to follow up with their physicians. Finally, additional training and resources, such as local community partners that could help connect DOs to healthcare providers, would be provided to clinics who participate in human-health-related programs.
  3. Extra time added to veterinary appointments: Depending on the DOs’ level of interest and engagement, as well as the VS availability at the clinic, time to conduct human biometric testing will vary. These appointments will be tailored to accommodate the PA needs of both the human and companion animal, so extra time, if warranted and desired, would be possible. Those DOs wishing to receive multiple health tests would likely require an additional appointment or a longer appointment time. In the study conducted by Duncan et al. [16], veterinary appointments were structured so that a technician conducted all biometric testing while the animal was with the veterinarian, thereby maintaining a similar appointment duration as appointments containing no human-health programming.
Top barriers reported by VS:
  1. Dog has medical issues: Although PA may not be appropriate for dogs with all types of medical issues, PA can be an important management component of and can help alleviate an array of ailments. Nevertheless, the prescription of PA must be handled on a case-by-case basis. Veterinarians can tailor the PA recommendations to each individual dog’s needs as they do for other treatment plans.
  2. Owner has a disability: In order to enroll in a PA program, participants must affirm that they acknowledge the risks (although minimal) associated with increased PA and certify that they are healthy enough to participate. Considerations for owner participation and ability will be accounted for as well.
  3. Client receptivity: In order to determine interest, individual clinics would have to survey their clientele. Clinics can develop a system to allow DOs to opt in or opt out of a PA-prescription program that respects potential concerns about discussing PA directly. Results of this study suggest that many clients would be interested in participating in this type of service; therefore, disseminating this information and empowering clinic staff could result in a significant public health benefit.

Although the benefits of dog-walking for human and companion animal health are clear, dog-walking interventions also have the potential to benefit environmental health [23,28]. Research indicates that humans who feel more connected to nature are more likely to engage in various pro-environmental behaviors, such as recycling or walking instead of driving [29,30]. Although the literature is inconclusive regarding whether time spent in nature (in this case via dog-walking) increases pro-environmental behavior directly or through nature connectedness, some evidence suggests that repeated exposure to nature does result in increased connection and subsequent pro-environmental behaviors [29]. Through dog-walking, DOs exposure to nature is increased significantly and consistently when compared to non-DOs. One Health is a concept that human, animal, and environmental health are interrelated [23]. Proponents of One Health suggest that through transdisciplinary collaboration, researchers can work to develop health-promotion interventions that simultaneously benefit all three health components [23]. Although more work is needed, dog-walking interventions have the potential to advance a One Health agenda by concurrently enhancing human, animal, and environmental health.

### 4.1. Strengths and Limitations

Strengths of this survey include the large sample size and wide geographic range of respondents from every state in the U.S. and Washington, DC. Limitations include restricting responses only to American DOs and VS. The collection of DO data via Amazon Mechanical Turk (MTurk) presents both strengths and limitations. Although data collected from MTurk are more diverse than data collected via other online mechanisms, research indicates that data collected from this system can show a positivity bias, as those who agree to participate are already interested in the subject area and therefore are more enthusiastic about the topic compared to non-participants [31]. Additionally, although data were collected anonymously, it is possible that participants responded in what they perceived to be a socially desirable manner (for example, reporting more favorable attitudes towards veterinary-prescribed PA). Lastly, because our DO sample was more likely to have a college education (47% of DOs had a bachelor’s degree compared with 36% among American adults [32]), the present results may not represent the willingness of all American DOs to participate in veterinary-prescribed PA programming. There is a positive correlation between rates of PA and educational attainment [33], so the average member of our sample may have been somewhat more inclined to PA participation than the average American. It is unclear whether similar results would be obtained in other countries or with a different DO population. In addition, some results of the DO and VS surveys could not be directly compared to one another because slightly different questions were asked; however, the study was not intended to be a comparison of veterinary vs. DO views on PA or PA programming, but instead these surveys were designed to independently assess how interested these two groups would be in such programs. The surveys were designed with that goal in mind, as well as with the goal of creating brief surveys that would be completed by many respondents. Due to the brief and independent nature of the surveys, some questions included in one survey were not included in the other. The survey questions that were compared between groups were asked identically in both groups. Finally, DOs were not asked if they were the primary caregiver for their dog, which could impact PA adherence and generalizability of the study results.

### 4.2. Future Directions

These results reveal that veterinary-prescribed PA programs such as the one conducted by Duncan and colleagues [16] would find receptive audiences among many private-practice veterinary clinics throughout the U.S. Further, many DOs would be interested in participating in such programs to benefit their own health as well as the health of their companion animals. Conducting such interventions at veterinary private practices appears to be a promising future direction; however, additional work is needed to address real or perceived barriers reported by VS. For example, VS would benefit from logistical and legal guidance around implementing such a program in their clinic, including information regarding the dissemination of confidential test results and taking care to only measure basic human biometrics, such as weight and blood pressure. VS should not attempt to interpret results, diagnose, or treat DOs without a proper license. Additionally, although walking is a safe and accessible form of PA for nearly all individuals [34], future studies should seek to address how safety concerns and elements of the built environment (such as streetlamps or sidewalks) impact veterinary-prescribed PA adoption and adherence. Lastly, this work could further be extended to determine the impact of a veterinary-prescribed PA intervention with other companion animals such as horses or cats.

## 5. Conclusions

Extending the veterinary-prescribed PA programming from settings in veterinary teaching hospital to private-practice veterinary clinics seems to be acceptable to both DOs and VS. In addition, many VS believe such programming would be feasible at their clinics; thus, pursuing such programs appears to be a promising avenue for promoting human and animal health.

## Figures and Tables

**Table 1:** Dog owner (DO; n = 1028) and veterinary staff (VS; n = 631) beliefs about the usefulness of physical activity (PA) for preventing disease.

Disease Type	Participant Rating of Usefulness for PA to Prevent Disease							
	**Both Humans and Dogs**		**Humans Only**		**Dogs Only**		**Neither Humans nor Dogs**	
	VS	DO	VS	DO	VS	DO	VS	DO
Obesity	98.3%	83.3%	0.8%	8.7%	0.6%	6.0%	0.3%	2.0%
Cardiovascular Disease	84.2%	79.7%	15.3%	10.6%	0.2%	7.4%	0.3%	2.3%
Cancer	61.0%	65.7%	6.8%	17.5%	0.5%	4.4%	31.7%	15.2%
Musculoskeletal Disease (arthritis, mobility, etc.)	98.0%	81.5%	1.0%	6.0%	1.0%	8.1%	0.0%	4.4%
High Blood Pressure	73.1%	69.7%	23.6%	23.2%	0.3%	5.5%	3.0%	4.5%
Mental Illness/ Behavior/Depression	92.9%	69.9%	6.3%	20.6%	0.3%	5.4%	0.5%	4.1%
Diabetes	84.5%	66.3%	10.9%	23.9%	0.2%	4.8%	4.4%	5.0%

**Table 2:** Dog owner (DO; n = 1028) and veterinary staff (VS; n = 631) ratings of the percent of people and dogs that would benefit from increased PA.

Participant Ratings	Percent of People and Dogs That Would Benefit from Increased PA					
	**0%**	**1–25%**	**26–50%**	**51–75%**	**76–99%**	**100%**
VS (People)	0.0	0.0	3.3	25.7	43.9	27.1
DO (People)	0.3	0.9	4.9	18.6	39.4	36.0
VS (Dogs)	0.0	0.2	5.7	25.8	42.6	25.7
DO (Dogs)	0.3	2.2	11.3	19.9	30.1	36.2

**Table 3:** Dog owner (DO; n = 1028) and veterinary staff (VS; n = 722) ratings of the frequency with which PA is discussed during veterinary visits.

Frequency	Participant Ratings of the Frequency of PA Discussions	
	DO	VS
Never (at NO appointments)	14.5%	3.5%
Sometimes (at FEW appointments)	42.1%	27.7%
Often (at ABOUT HALF of appointments)	20.4%	21.1%
Usually (at MOST appointments)	17.9%	27.4%
Always (at EVERY appointment)	5.1%	13.2%

**Table 4:** Dog owners’ (n = 1028) self-reported comfort of having biomarkers measured at a veterinary clinic.

Biomarker	Participant Level of Comfort			
	**Very** **Comfortable**	**Somewhat** **Comfortable**	**Somewhat Uncomfortable**	**Very** **Uncomfortable**
Height	65.5%	20.9%	7.3%	6.3%
Weight	53.6%	25.7%	12.4%	8.4%
Body Mass Index (BMI)	51.5%	26.5%	13.6%	8.5%
Waist Circumference	47.1%	28.2%	15.2%	9.5%
Blood test (via finger prick)	43.3%	26.8%	18.0%	11.9%
Blood pressure	55.5%	25.8%	10.5%	8.2%

**Table 5:** Reasons why dog owners (n = 1028) would participate in joint human–animal health activities.

Reason	Percent Agreeing
Convenience: It could be convenient to have health screenings performed at the same location for me and my dog(s).	51.5%
Cost: I would be likely to participate if this service was more affordable than general health screenings at other facilities.	45.4%
Education: I might pursue this as an opportunity to increase my knowledge about topics important to both my health and my dog’s health.	42.7%
Trust: I trust my veterinarian and veterinary staff.	42.6%
Awareness: Test results (from the vet clinic) may make me more likely to get an appointment with my doctor.	35.1%
Stress: It may be less stressful to have the health-screening activities done at my veterinary clinic relative to other options.	24.4%
Frequency: I would get my general health screening done more often if I could do it at my veterinary clinic instead of or in addition to at a human healthcare center.	24.1%

**Table 6:** Reasons why dog owners (n = 1028) would not participate in joint human–animal health activities.

Reason	Percent Agreeing
Cost: These services might increase the cost of my veterinary visit.	54.1%
Training: Veterinary staff are not trained in, or experts in, human health.	29.8%
Time: There is not enough time during my veterinary appointments for additional services.	29.0%
Need: I already regularly get general health screenings elsewhere.	26.9%
Comfort: I would not feel comfortable with the veterinary staff knowing about my general health.	25.9%
Credibility: I do not know that results obtained at a veterinary clinic would be deemed credible by my medical doctor.	23.3%
Mission: I do not want the staff at my veterinary clinic to be distracted or diverted from their mission of promoting animal health.	19.3%
None: None of these factors would keep me from participating.	5.5%

**Table 7:** Perceived barriers that veterinary staff (n = 631) believe reduce their likelihood of discussing physical activity during appointments.

Barrier	Percent Agreeing
The pet has other medical issues that are more important to address than physical activity.	62.3%
The client is unable to increase physical activity with their pet due to a physical or mental disability.	54.4%
The client is often not receptive to discussing physical activity for their pet	37.7%
There is not enough time during most appointments to discuss physical activity unless critical to the patient’s health.	33.6%
Owner compliance with physical activity recommendation is too low to make it worthwhile.	21.2%
It is awkward to discuss weight-related topics with clients.	20.1%
I should increase my own and/or my pets’ physical activity, and I feel hypocritical recommending it.	15.4%
Recommending physical activity might make the owner uncomfortable and keep them from coming back to our clinic.	8.9%
Other	4.6%

**Table 8:** Resources veterinary staff (n = 631) believe would help them promote healthy behaviors for their clients and pets.

Resource	Percent Agreeing
Complete toolkit: A collection of materials that a practice could use to initiate physical activity programming in their clinic.	77.3%
Education: Information regarding the health benefits of increasing physical activity for both animals and people.	69.1%
Physical resources: Information to provide clients about the health benefits of physical activity or other health-promoting behaviors.	66.4%
Communication tools: Strategies to discuss physical activity with my clients.	61.7%
Legal support: Information regarding the legal boundaries of discussing health topics with clients that do not directly pertain to the health of their pet.	28.1%

## Data Availability

The data presented in this study are openly available in the Open Science Framework at https://doi.org/10.17605/OSF.IO/PBT5R (accessed on 18 February 2021).

## Supplementary Materials

The following are available online at https://www.mdpi.com/1660-4601/18/5/2339/s1, Appendix A: dog owner survey, Appendix B: veterinary survey, located at https://doi.org/10.17605/OSF.IO/PBT5R (accessed on 18 February 2021).

## References

[1] Physical Activity. https://www.who.int/news-room/fact-sheets/detail/physical-activity.

[2] HealthyAtHome—Physical Activity. https://www.who.int/news-room/campaigns/connecting-the-world-to-combat-coronavirus/healthyathome/healthyathome---physical-activity#:~:text=All%20adults%20should%20do%20at,physical%20activity%20throughout%20the%20week.

[3] Walk. Run. Dance. Play. What’s Your Move?—Move Your Way | Health.Gov. https://health.gov/moveyourway.

[4] Community-Wide Campaigns | Active People, Healthy Nation | Physical Activity | CDC. https://www.cdc.gov/physicalactivity/activepeoplehealthynation/strategies-to-increase-physical-activity/community-wide-campaigns.html.

[5] Thornton J.S., Frémont P., Khan K., Poirier P., Fowles J., Wells G.D., Frankovich R.J. (2016). Physical Activity Prescription: A Critical Opportunity to Address a Modifiable Risk Factor for the Prevention and Management of Chronic Disease: A Position Statement by the Canadian Academy of Sport and Exercise Medicine. Br. J. Sports Med..

[6] Fletcher Gerald F., Carolyn L., Josef N., Cemal O., Ross A., Lavie Carl J. (2018). Promoting Physical Activity and Exercise. J. Am. Coll. Cardiol..

[7] Oberg E. (2007). Physical Activity Prescription: Our Best Medicine. Integr. Med..

[8] Kallings L.V., Leijon M., Hellénius M.-L., Ståhle A. (2007). Physical Activity on Prescription in Primary Health Care: A Follow-up of Physical Activity Level and Quality of Life. Scand. J. Med. Sci. Sports.

[9] Whitsel L.P., Bantham A., Jarrin R., Sanders L., Stoutenberg M. (2020). Physical Activity Assessment, Prescription and Referral in US Healthcare: How Do We Make This a Standard of Clinical Practice?. Prog. Cardiovasc. Dis..

[10] Maiorana A., Levinger I., Davison K., Smart N., Coombes J. (2018). Exercise Prescription Is Not Just for Medical Doctors: The Benefits of Shared Care by Physicians and Exercise Professionals. Br. J. Sports Med..

[11] Thornton J. (2018). Physical Activity Prescription and Engaging the Entire “Community of Practice”. Br. J. Sports Med..

[12] Buckley B.J.R., Finnie S.J., Murphy R.C., Watson P.M. (2020). “You’ve Got to Pick Your Battles”: A Mixed-Methods Investigation of Physical Activity Counselling and Referral within General Practice. Int. J. Environ. Res. Public. Health.

[13] Pollak K.I., Coffman C.J., Alexander S.C., Østbye T., Lyna P., Tulsky J.A., Bilheimer A., Dolor R.J., Lin P.H., Bodner M.E. (2014). Weight’s up? Predictors of Weight-Related Communication during Primary Care Visits with Overweight Adolescents. Patient Educ. Couns..

[14] Doctors Don’t Have to Dread Discussing Dieting | ACP Internist. https://acpinternist.org/archives/2012/11/diet.htm.

[15] Teixeira M.E., Budd G.M. (2010). Obesity Stigma: A Newly Recognized Barrier to Comprehensive and Effective Type 2 Diabetes Management. J. Am. Acad. Nurse Pract..

[16] Duncan C., Carswell A., Nelson T., Graham D.J., Duerr F.M. (2020). Veterinary-Prescribed Physical Activity Promotes Walking in Healthy Dogs and People. BMC Vet. Res..

[17] Rhodes R.E., Baranova M., Christian H., Westgarth C. (2020). Increasing Physical Activity by Four Legs Rather than Two: Systematic Review of Dog-Facilitated Physical Activity Interventions. Br. J. Sports Med..

[18] Westgarth C., Christley R.M., Christian H.E. (2014). How Might We Increase Physical Activity through Dog Walking?: A Comprehensive Review of Dog Walking Correlates. Int. J. Behav. Nutr. Phys. Act..

[19] Westgarth C., Christley R.M., Jewell C., German A.J., Boddy L.M., Christian H.E. (2019). Dog Owners Are More Likely to Meet Physical Activity Guidelines than People without a Dog: An Investigation of the Association between Dog Ownership and Physical Activity Levels in a UK Community. Sci. Rep..

[20] Higgins J.W., Temple V., Murray H., Kumm E., Rhodes R. (2013). Walking Sole Mates: Dogs Motivating, Enabling and Supporting Guardians’ Physical Activity. Anthrozoös.

[21] Kushner R.F., Blatner D.J., Jewell D.E., Rudloff K. (2006). The PPET Study: People and Pets Exercising Together. Obesity.

[22] Allen K., Blascovich J., Mendes W.B. (2002). Cardiovascular Reactivity and the Presence of Pets, Friends, and Spouses: The Truth about Cats and Dogs. Psychosom. Med..

[23] Yuma P., Fowler J., Duerr F., Kogan L., Stockman J., Graham D.J., Duncan C. (2019). Promoting Outdoor Physical Activity for People and Pets: Opportunities for Veterinarians to Engage in Public Health. Top. Companion Anim. Med..

[24] Bauman A.E., Russell S.J., Furber S.E., Dobson A.J. (2001). The Epidemiology of Dog Walking: An Unmet Need for Human and Canine Health. Med. J. Aust..

[25] Bauman A., Christian H., Thorpe R.J.J., Macniven R. (2011). International Perspectives on the Epidemiology of Dog Walking. The Health Benefits of Dog Walking for People and Pets: Evidence and Case Studies.

[26] Byers C.G., Wilson C.C., Stephens M.B., Goodie J.L., Netting F.E., Olsen C.H. (2014). Owners and Pets Exercising Together: Canine Response to Veterinarian-Prescribed Physical Activity. Anthrozoös.

[27] Census of Veterinarians Finds Trends with Shortages, Practice Ownership. https://www.avma.org/javma-news/2019-07-15/census-veterinarians-finds-trends-shortages-practice-ownership.

[28] Colléony A., White R., Shwartz A. (2019). The Influence of Spending Time Outside on Experience of Nature and Environmental Attitudes. Landsc. Urban Plan..

[29] Martin L., White M.P., Hunt A., Richardson M., Pahl S., Burt J. (2020). Nature Contact, Nature Connectedness and Associations with Health, Wellbeing and pro-Environmental Behaviours. J. Environ. Psychol..

[30] Alcock I., White M.P., Pahl S., Duarte-Davidson R., Fleming L.E. (2020). Associations between Pro-Environmental Behaviour and Neighbourhood Nature, Nature Visit Frequency and Nature Appreciation: Evidence from a Nationally Representative Survey in England. Environ. Int..

[31] Matherly T. (2018). A Panel For Lemons? Positivity Bias, Reputation Systems and Data Quality on MTurk. Eur. J. Mark..

[32] U.S. Census Bureau Releases New Educational Attainment Data. https://www.census.gov/newsroom/press-releases/2020/educational-attainment.html.

[33] Adults That Met Physical Activity Guidelines by Education U.S. 2008–2017 | Statista. https://www.statista.com/statistics/1032924/share-of-us-population-that-meets-physical-activity-guidelines-by-education/.

[34] Fogelholm M. (2005). Walking for the Management of Obesity. Dis. Manag. Health Outcomes.

