# Supplementary material for: Veterinary-Prescribed Physical Activity: Feasibility and Acceptability among Veterinary Staff and Dog Owners
Source: Int J Environ Res Public Health. 2021 Feb 27;18(5):2339. doi: 10.3390/ijerph18052339 (PMC7967723; doi:10.3390/ijerph18052339)
Supplement: Supplementary file 1 [file ijerph-18-02339-s001.pdf]

Appendix A

Dog Owner Survey

Q1 This research is being conducted by faculty members at Colorado State University. The goal of this survey is to better understand dog owner attitudes and behaviors related to physical activity for both dog and dog-owner health.

If you are at least 18 years old and a dog owner, you are invited to complete a short anonymous online survey for which you will be paid $2. Only one respondent per IP address will be compensated. Your participation in this research is voluntary. We will not collect your name or personal identifiers and when we share the data with others, we will combine the data from all participants. There are no direct benefits or known risks associated with participation in this survey.

We anticipate the survey will take 5-10 minutes to complete.

If you have any questions about the research, please contact Dr. Dan Graham at (dan.graham@colostate.edu). If you have any questions about your rights as a volunteer in this research, contact the CSU IRB at: RICRO_IRB@mail.colostate.edu; 970-491-1553.

Q2 How many dogs do you own currently?

- ○ 0
- ○ 1
- ○ 2
- ○ 3 or more

Q3 Thank you for your interest, but we are only surveying dog owners at this time.

Q4 What is your age, in years?

▼ 18 ... over 100 years

Q5 What is your gender?

- ◯ Male
- ◯ Female
- ◯ Other or prefer not to answer

Q6 What is the highest level of education you have completed?

- ◯ Less than high school degree
- ◯ High school degree or equivalent (e.g., GED)
- ◯ Some college but no degree
- ◯ Associate's Degree
- ◯ Bachelor's Degree
- ◯ Graduate or professional degree
- ◯ Other (please list) ____

Q7 In which zip code do you currently live?

____

Q8 How important do you believe regular physical activity is for **DOG** health?

- Extremely important
- Very important
- Moderately important
- Slightly important
- Not at all important

Q9 How important do you believe regular physical activity is for **HUMAN** health?

- Extremely important
- Very important
- Moderately important
- Slightly important
- Not at all important

Q10 For each of the following conditions, please indicate whether you consider physical activity to be useful for prevention and/or treatment of the condition in humans, dogs, both humans and dogs, or neither humans nor dogs.

	Humans	Dogs	Both	Neither
Obesity	○	○	○	○
Cardiovascular Disease	○	○	○	○
Cancer	○	○	○	○
Musculoskeletal Disease (arthritis, mobility, etc.)	○	○	○	○
High Blood Pressure	○	○	○	○
Mental Illness / Behavior / Depression	○	○	○	○
Diabetes	○	○	○	○

Q11 What percent of people and dogs do you believe would benefit from increasing their physical activity?

	0%	1-25%	26-50%	51-75%	76-99%	100%
People	○	○	○	○	○	○
Dogs	○	○	○	○	○	○

Q12 Estimate the average time in minutes per day that you spend doing physical activity (anything that gets your heart beating faster).

- None (0 minutes/day)
- 1-15 min/day
- 16-30 min/day
- 31-45 min/day
- 46-60 min/day
- 61-75 min/day
- 76-90 min/day
- >90 min/day

Q13 What percent of the daily physical activity you reported above is spent with your dog(s)?

- None (0%)
- 1-20%
- 21-40%
- 41-60%
- 61-80%
- >80%

Q14 How often does your veterinarian talk with you about the importance of physical activity for promoting your dog(s) health?

- Never (at NO appointments)
- Sometimes (at FEW appointments)
- Often (at ABOUT HALF of appointments)
- Usually (at MOST appointments)
- Always (at EVERY appointment)

Q15 If your veterinarian told you that increasing your dog(s)' physical activity was important for your dog(s)' health, what would you do?

- I would make sure my dog was more active EVERY day
- I would make sure my dog was more active MOST days
- I would make sure my dog was more active SOME days
- I would not increase my dog’s activity

Q16 Do you believe increasing your dog(s)' physical activity would also increase your own level of physical activity?

- Definitely yes
- Probably yes
- Not sure
- Probably no
- Definitely no

Q17 Why or why not?

____

Q18 At Colorado State University (CSU), a pilot walking-prescription program targeted dog-human pairs not meeting physical activity recommendations (150 min/week). In addition to a weekly exercise goal and logbook, both dogs and people received pre- and post-program body assessments (height, weight, waist circumference, and Body Mass Index (BMI)) and basic bloodwork done using a simple finger prick by a trained and certified technician. This program was feasible, well-received, and effective at increasing physical activity. An unexpected benefit was that the human health screenings done at the veterinary clinic identified previously unknown, modifiable risk factors or disease conditions when the screening test results were evaluated by the dog owners' medical doctor. While this program was successful, the research team believes that academic veterinary hospitals may not be the only venue for such health screening activities. The following questions are designed to investigate the potential for dog owners to participate in similar human/animal health-promoting activities at private-practice veterinary clinics.

Q19 Would you be willing to participate in health screening activities at your veterinary clinic like those included in the study described above?

- ○ Definitely yes
- ○ Probably yes
- ○ Not sure
- ○ Probably no
- ○ Definitely no

Q20 Why or why not?

____

Q21 How comfortable would you be having each of the following measured at a veterinary clinic by an appropriately trained and certified staff member?

	Very comfortable	Somewhat comfortable	Somewhat uncomfortable	Very uncomfortable
Height	○	○	○	○
Weight	○	○	○	○
Body Mass Index (BMI)	○	○	○	○
Waist Circumference	○	○	○	○
Blood test (via finger prick)	○	○	○	○
Blood pressure	○	○	○	○

Q22 Which of the following general health promotion topics that apply to both humans and animals would you be interested in learning more about at a veterinary clinic? Select all that apply.

- ☐ Healthy eating / nutrition
- ☐ Weight loss and treatment for obesity
- ☐ Diseases that can be spread between animals and people
- ☐ Antibiotic resistance
- ☐ Sleep
- ☐ Stress/mental health
- ☐ Spending time outdoors
- ☐ Physical activity
- ☐ Other (please list) ____

Q23

The reason(s) I might participate in joint human-and-animal health activities described above at a private practice veterinary clinic include (select all that apply):

☐ Convenience: It could be convenient to have health screenings performed at the same location for me and my dog(s)

☐ Trust: I trust my veterinarian and veterinary staff.

☐ Stress: It may be less stressful to have the health screening activities done at my veterinary clinic relative to other options.

☐ Cost: I would be likely to participate if this service was more affordable than general health screenings at other facilities.

☐ Frequency: I would get my general health screening done more often if I could do it at my veterinary clinic instead of or in addition to at a human health care center

☐ Education: I might pursue this as an opportunity to increase my knowledge about topics important to both my health and my dog's health.

☐ Awareness: Test results (from the vet clinic) may make me more likely to get an appointment with my doctor

☐ Other (please list): ____

☐ None: None of these factors would matter. I am not interested in participating in such a program.

Q24 The reasons I might NOT participate in joint human-and-animal health activities described above at a private practice veterinary clinic include (select all that apply):

☐ Cost: These services might increase the cost of my veterinary visit.

☐ Comfort: I would not feel comfortable with the veterinary staff knowing about my general health.

☐ Need: I already regularly get general health screenings elsewhere.

☐ Time: There is not enough time during my veterinary appointments for additional services.

☐ Mission: I don't want the staff at my veterinary clinic to be distracted or diverted from their mission of promoting animal health.

☐ Training: Veterinary staff are not trained in, or experts in, human health.

☐ Credibility: I don't know that results obtained at a veterinary clinic would be deemed credible by my medical doctor.

☐ Other (please list): ____

☐ None: None of these factors would keep me from participating

---

Q25 As you have seen, we are interested in finding ways veterinarians can help improve the health of human dog owners without a lot of extra work for them. Are there other ways that you think vets could help to simultaneously promote the health of humans and their pets?

____

Q26 Thank you for completing our survey!
This is the code you can enter into MTurk to receive compensation: FKPDP8741H

Appendix B

Veterinary Survey
Q1 This research is being conducted by faculty members at Colorado State University. The goal of this survey is to better understand veterinarians' attitudes and behaviors related to physical activity for both animal and owner health.
You are invited to complete a short anonymous online survey. Your participation in this research is voluntary. We will not collect your name or personal identifiers and when we share the data with others, we will combine the data from all participants. There are no direct benefits or known risks associated with participation in this survey.
We anticipate the survey will take 5-10 minutes to complete. If you work in veterinary medicine and you are one of the first 200 people to complete the survey, you will be eligible to enter a raffle to win 1 of 10 $50 Amazon gift cards.
If you have any questions about the research, please contact Dr. Colleen Duncan at (colleen.duncan@colostate.edu). If you have any questions about your rights as a volunteer in this research, contact the CSU IRB at: RICRO_IRB@mail.colostate.edu; 970-491-1553.

Q2 Which of the following best describes your role in veterinary medicine?

- ○ Veterinarian
- ○ Veterinary Technician
- ○ Client Services (Reception, Accounting, Administration)
- ○ Veterinary Assistant
- ○ Other ____
- ○ I do not work in veterinary medicine

Q3 In which state do you primarily work?

- ○ Alabama
- ○ Alaska
- ○ Arizona
- ○ Arkansas
- ○ California
- ○ Colorado
- ○ Connecticut
- ○ Delaware
- ○ Florida
- ○ Georgia
- ○ Hawaii
- ○ Idaho
- ○ Illinois
- ○ Indiana
- ○ Iowa
- ○ Kansas
- ○ Kentucky
- ○ Louisiana
- ○ Maine
- ○ Maryland

- Massachusetts
- Michigan
- Minnesota
- Mississippi
- Missouri
- Montana
- Nebraska
- Nevada
- New Hampshire
- New Jersey
- New Mexico
- New York
- North Carolina
- North Dakota
- Ohio
- Oklahoma
- Oregon
- Pennsylvania
- Rhode Island
- South Carolina
- South Dakota

- Tennessee
- Texas
- Utah
- Vermont
- Virginia
- Washington
- West Virginia
- Wisconsin
- Wyoming
- Washington D.C.
- Other (Please Specify): ____

Q4 In which zip code do you work primarily?

____

Q5 Which category includes your age?

- 20-29 years
- 30-39 years
- 40-49 years
- 50-59 years
- 60-69 years
- 70+ years

Q6 For the majority of my career, I have predominantly worked in...

- Small animal clinical practice
- Mixed animal clinical practice
- Large animal clinical practice
- Academia
- Other (Please Describe) ____

Q7 For what percent of your patients are you their primary care provider?

-
- 26-50%
- 51-75%
- >75%

Q8 How important do you believe regular physical activity is for **DOG** health?

- Extremely important
- Very important
- Moderately important
- Slightly important
- Not at all important

Q9 How important do you believe regular physical activity is for **HUMAN** health?

- Extremely important
- Very important
- Moderately important
- Slightly important
- Not at all important

Q10 For each of the following conditions, please indicate whether you consider physical activity to be useful for prevention and/or treatment of the condition in humans, dogs, both humans and dogs, or neither humans nor dogs.

	Humans	Dogs	Both	Neither
Obesity	○	○	○	○
Cardiovascular Disease	○	○	○	○
Cancer	○	○	○	○
Musculoskeletal Disease (arthritis, mobility, etc.)	○	○	○	○
High Blood Pressure	○	○	○	○
Mental Illness / Behavior / Depression	○	○	○	○
Diabetes	○	○	○	○

Q11 What percent of people and dogs do you believe would benefit from increasing their physical activity?

	0%	1-25%	26-50%	51-75%	76-99%	100%
People	○	○	○	○	○	○
Dogs	○	○	○	○	○	○

Q12 How often do you ask about the patient's physical activity level when you take a history?

- Never (at NO appointments)
- Sometimes (at FEW appointments)
- Often (at ABOUT HALF of appointments)
- Usually (at MOST appointments)
- Always (at EVERY appointment)
- Not Applicable (I do not take histories)

Q13 For what percent of patients do you recommend additional physical activity?

- 0%
- 1-25%
- 26-50%
- 51-75%
- 76-99%
- 100%

Q14 When do you recommend increased physical activity for a patient? Select all that apply:

- ☐ When the patient is overweight or obese
- ☐ When the patient has a chronic illness (where activity is not contraindicated)
- ☐ When the patient has arthritis
- ☐ When the patient displays anxiety or other behavioral issues
- ☐ When the patient is healthy
- ☐ Other (please list) ____

Q15 Which of the following are barriers that reduce your likelihood of discussing physical activity during appointments? Select all that apply:

- ☐ It is awkward to discuss weight-related topics with clients.
- ☐ Owner compliance with physical activity recommendation is too low to make it worthwhile.
- ☐ The client is often not receptive to discussing physical activity for their pet.
- ☐ The pet has other medical issues that are more important to address than physical activity.
- ☐ The client is unable to increase physical activity with their pet due to a physical or mental disability.
- ☐ I should increase my own and/or my pets' physical activity, and I feel hypocritical recommending it.
- ☐ Recommending physical activity might make the owner uncomfortable and keep them from coming back to our clinic.
- ☐ There is not enough time during most appointments to discuss physical activity unless critical to the patient's health.
- ☐ Other (please list) ____

Q16 For what percent of clients/owners do you highlight the human health benefits of physical activity within the context of promoting physical activity for their pet?

- ○ 0%
- ○ 1-25%
- ○ 26-50%
- ○ 51-75%
- ○ 76-99%
- ○ 100%

Q17 At Colorado State University (CSU), a pilot walking prescription program is wrapping up and we are interested to know if similar programs would be feasible in private practices. The CSU program targeted dog-human pairs not currently meeting physical activity recommendations (150min/week). In addition to a weekly exercise goal and logbook, both dogs and people received pre and post body assessments and basic bloodwork. Preliminary data review suggests that this programing was feasible, well received and effective at increasing PA. An unexpected benefit was that the human health screenings done at the veterinary clinic (by a veterinary technician with training and appropriate permissions) identified previously unknown, modifiable risk factors or disease conditions when the results were evaluated by the dog owners' medical doctor.

While this program was successful, the research team believes that academic hospitals may not be the only venue for such initiatives. The following questions are designed to investigate the potential for private practice veterinarians to engage in similar human-animal health-promoting activities.

Q18 Which of the following resources might help veterinarians promote healthy behaviors for clients and their pets? Select all that apply:

- ☐ Physical resources: Information to provide clients about the health benefits of physical activity or other health promoting behaviors
- ☐ Communication tools: Strategies to discuss physical activity with my clients
- ☐ Legal support: Information regarding the legal boundaries of discussing health topics with clients that do not directly pertain to the health of their pet
- ☐ Education: Information regarding the health benefits of increasing physical activity for both animals and people
- ☐ Complete toolkit: A collection of the above information that a practice could use to initiate physical activity programming in their clinic

Q19 Regarding the inclusion of basic human health promotion services, as described above with emphasis on physical activity with a pet, please rate your agreement or disagreement with how each might benefit private veterinary practices.

	Strongly agree	Somewhat agree	Neither agree nor disagree	Somewhat disagree	Strongly disagree
Marketing: offering new and unique health-programs could be a promotional opportunity	○	○	○	○	○
Economics: health-promotion programs could increase practice revenue	○	○	○	○	○
Clinic culture: health-promotion programs could be enjoyable for clinic staff to conduct	○	○	○	○	○
Networking: health-promotion programs may provide new opportunities to work with other community health organizations	○	○	○	○	○

Patient health: It could improve the health of clinic patients because owners with healthy lifestyles (e.g., more physical activity) tend to have healthier pets	○	○	○	○	○
Other (please list)	○	○	○	○	○

Q20 Which client groups do you think would be receptive to receiving human health promotion information from their veterinarian? Select all that apply:

- ☐ Younger dog owners
- ☐ Senior dog owners
- ☐ Families with children
- ☐ Dog owners with lack of access to regular human health services
- ☐ Dog owners with chronic health conditions
- ☐ Other (please list) ____
- ☐ None, I don't think any of my clients would be receptive to this type of information

Q21 If a health promotion program were to be initiated in private practice, which member of the clinical team would be best suited to manage it?

- ○ Clinic owner
- ○ Veterinarian
- ○ Veterinary technician
- ○ Hospital manager
- ○ Position description is not important, the individual with the most interest and skills in this area should oversee such initiatives
- ○ Not a topic that should be discussed in a veterinary private practice setting.
- ○ Other (please list) ____

Q22 Which of the following are potential barriers to private veterinary practices offering some type of human-animal health promotion programming (with emphasis on physical activity)? Select all that apply:

- ☐ Lack of interest on part of clinic staff
- ☐ Lack of interest in the client population
- ☐ Legal concerns
- ☐ Economics of offering such a program
- ☐ Lack of time on the part of clinic staff
- ☐ Other (please list) ____

Q23 Which of the following general health promotion topics that apply to both humans and animals would be appropriate for veterinary clinic personnel to discuss with their clients? Select all that apply.

- ☐ Healthy eating / nutrition
- ☐ Weight loss and treatment for obesity
- ☐ Diseases that can be spread between animals and people
- ☐ Antibiotic resistance
- ☐ Sleep
- ☐ Stress/mental health
- ☐ Spending time outdoors
- ☐ Physical activity
- ☐ Other (please list) ____

Q24 How did you hear about this survey?

- ☐ DVM360 email
- ☐ DVM360 Facebook page
- ☐ From a friend/colleague
- ☐ Press release / media story
- ☐ Other (please list) ____

Q25 Would you, or others at your hospital, be interested in potentially participating in future initiatives on how to incorporate shared human-animal health initiatives in private practice?

- ○ Yes (please submit contact information upon completion of this survey)
- ○ Maybe, I'll keep my eyes open for opportunities to engage
- ○ No

Q26 Would you like to be entered to win one of 10 Amazon $50 gift certificates? If yes you will be redirected to another page to enter your contact information. A random drawing will be made from the first 100 respondents. Your contact information will not be associated with your responses to this questionnaire.

- ○ Yes
- ○ No
